# Experimental Models of Maternal Obesity and Neuroendocrine Programming of Metabolic Disorders in Offspring

**DOI:** 10.3389/fendo.2017.00245

**Published:** 2017-09-25

**Authors:** Clare M. Reynolds, Stephanie A. Segovia, Mark H. Vickers

**Affiliations:** ^1^Liggins Institute, University of Auckland, Auckland, New Zealand

**Keywords:** developmental programming, maternal nutrition, neuroendocrine, appetite regulation, epigenetics, metabolic syndrome

## Abstract

Evidence from epidemiological, clinical, and experimental studies have clearly shown that disease risk in later life is increased following a poor early life environment, a process preferentially termed developmental programming. In particular, this work clearly highlights the importance of the nutritional environment during early development with alterations in maternal nutrition, including both under- and overnutrition, increasing the risk for a range of cardiometabolic and neurobehavioral disorders in adult offspring characterized by both adipokine resistance and obesity. Although the mechanistic basis for such developmental programming is not yet fully defined, a common feature derived from experimental animal models is that of alterations in the wiring of the neuroendocrine pathways that control energy balance and appetite regulation during early stages of developmental plasticity. The adipokine leptin has also received significant attention with clear experimental evidence that normal regulation of leptin levels during the early life period is critical for the normal development of tissues and related signaling pathways that are involved in metabolic and cardiovascular homeostasis. There is also increasing evidence that alterations in the epigenome and other underlying mechanisms including an altered gut–brain axis may contribute to lasting cardiometabolic dysfunction in offspring. Ongoing studies that further define the mechanisms between these associations will allow for identification of early risk markers and implementation of strategies around interventions that will have obvious beneficial implications in breaking a programmed transgenerational cycle of metabolic disorders.

## Introduction

Evidence from clinical and experimental studies has linked a maternal obesogenic environment to increase in the risk for the development of obesity and related cardiometabolic disorders in offspring in adulthood. As such, although there is a genetic component to obesity risk, environmental influences and gene–environment interactions are seen as primary mediators in the etiology of the current obesity epidemic (1, 2). Many of the perinatal exposures examined experimentally are associated with reorganization of neurodevelopmental pathways that play a role in energy expenditure and appetite regulation in ways that increase the later risk for obesity and related metabolic dysfunction in offspring (3). Although a consensus has been reached that an obesogenic environment in the periconceptual, pregnancy and lactation periods increases the risk of metabolic disorders in adulthood, the potential mechanisms that confer this increased risk are less well defined (4). One of the most investigated (and experimentally consistent) mechanisms is around that of altered development of the hypothalamus during critical periods of developmental plasticity. Evidence from a range of experimental model species has now shown that changes in the maternal diet can lead to alterations in the wiring of the neuroendocrine pathways in the hypothalamus that control energy balance and appetite regulation in offspring. Further, a number of key hormonal factors have been identified that may mediate early developmental malprogramming by maternal overnutrition including insulin, leptin and ghrelin. In this context, maternal obesity can modify blood–brain barrier (BBB) functionality with altered organization and permeability to such factors directed toward the arcuate nucleus of the mediobasal hypothalamus (ARH) (5). Obesity is a common characteristic observed in offspring born to high-fat diet (HFD)-fed mothers concomitant with hyperphagia that reflects altered hypothalamic development and related changes in the expression of these key neuropeptides (6). Both leptin (7–9) and insulin (10–12) are strongly neurotrophic, so alterations in availability of either during the period of developmental plasticity can underpin some of these adverse developmental changes. Under normal conditions, when adipose stores increase, leptin and insulin levels increase with activation of proopiomelanocortin (POMC) neurons and subsequent inhibition of neuropeptide Y (NPY) and agouti-related peptide (AgRP), thus providing a negative feedback loop that regulates intake and prevents obesity. This feedback system is perturbed following adverse developmental programming [i.e., the developmental origins of health and disease (DOHaD)] with alterations in ARC wiring appearing to be relatively conserved across model species. There is also a growing body of evidence that suggests a critical role for neuroimmune interactions during the prenatal period in the programming of neurobehavioral disorders in postnatal life (13).

Animal models allow controlled investigation of the interactions between genetics and early development and subsequent effects of early life perturbations on the programming of neural pathways which control the regulation of energy balance. A primary focus of early DOHaD experimental work was *via* models that utilized a range of maternal nutritional deficiencies (global or macronutrient imbalance including low protein diets) to induce fetal growth restriction. However, given the current obesogenic environment, there has been a move toward models that investigate the impact of maternal and/or neonatal overnutrition on programming of alterations in energy balance and appetite regulation in offspring. As with the maternal undernutrition models, a number of studies across a number of species and different overnutrition exposures have now similarly shown that an early life obesogenic environment leads to altered development of the central pathways that regulate appetite control and energy balance. Whether the mechanisms are the same remains to be elucidated but maternal intakes at both ends of the dietary spectrum are characterized by offspring obesity and metabolic disorders. This review will cover the range of experimental models of maternal obesity currently used, the nature of the early life exposures examined, potential commonalities in mechanisms and potential limitations of some of the approaches used.

## Experimental Models of Maternal Obesity

A number of experimental models of maternal obesity have been developed across a range of model species, dietary compositions, and duration of exposures. Programmed outcomes are relatively conserved across the models used and include impaired insulin sensitivity, hypertension and endothelial dysfunction, increased adiposity (with hyperleptinemia), and altered appetite regulation (e.g., hyperphagia) (6). Overlaid on this are sex-specific effects, which to date have not been well characterized, and long-term transgenerational impacts (14).

Logistical considerations can be a primary driver for the choice of animal model and therefore rodents are the most commonly used due to short gestational length, short timeframe to maturity of offspring and the opportunity to examine transgenerational effects and sexually dimorphic responses to programming stimuli. Rodents also have the benefit of being easily manipulated genetically and use of targeted knockouts, for example, to elucidate mechanisms. Use of models in larger animals including the sheep, pig, or non-human primate (NHP), although potentially having the better translatability to the human condition due to developmental timing (and being particularly conducive to fetal studies), have limitations around cost, gestational length and time to offspring maturity.

### Rodents

Most work to date has been undertaken in the rodent, primarily the rat. As above, although the rodent has some distinct logistical and cost advantages over other model species (short gestation length/lactation period, utility in transgenerational studies, and relative ease in manipulating genetically), the critical developmental windows in early life where the pathways that regulate energy balance and appetite control are set, differ significantly between humans and rodents (15). In the rodent, the development of neuronal pathways occurs early in the neonatal period while humans and other large model species these pathways develop in fetal life (16). Despite these differences in developmental windows, outcomes from rodent studies closely parallel those observations made in maternal obesity models in larger animals including sheep, swine, and NHP.

During early postnatal development in the rodent, axon outgrowth from ARH neurons is stimulated by leptin, and preautonomic NPY/AgRP/gamma-aminobutyric acid neurons are particularly sensitive to the known neurotrophic effects of leptin (17). Early work by Bouret et al. highlighted the trophic role of neonatal leptin in mediating the process of neurite outgrowth that eventually form the projections from the ARH to the paraventricular nucleus (PVN) and other areas of the hypothalamus (7). This window of action was specific to the neonatal period; neonatal leptin administration to leptin deficient *ob/ob* mice normalized wiring of the ARH, whereas treatment in the postweaning period had no effect. A number of studies have now similarly shown that maintenance of a “critical” leptin threshold during the early life period is required to facilitate normal tissue development and signaling pathways that are involved in metabolic homeostasis. In the rat, maternal obesity can lead to a period of neonatal hypoleptinemia and hypoinsulinemia although adulthood in these animals is characterized by obesity and insulin/leptin resistance (18). This may be due to abnormalities in the neonatal leptin surge as regards both amplitude and timing with work by Kirk et al. showing that rat neonates born to obese mothers later display an abnormal and prolonged neonatal leptin surge (19). It also has been recently shown in mice that neonatal insulin action leads to impaired hypothalamic neurocircuit formation in response to a maternal HFD (20). Targeted abrogation of insulin signaling specific to POMC neurons of offspring of HFD fed mothers restored POMC innervation of preautonomic PVH neurons and corrected the impaired glucose tolerance otherwise seen in these animals.

It has been shown that maternal and postnatal overnutrition (induced *via* a reduction in litter size) can differentially impact on appetite regulation and energy metabolism (21). Maternal obesity has been linked to hyperlipidemia in offspring paralleled by increased hypothalamic POMC mRNA and reduced NPY expression; these changes being amplified in offspring in the presence of a postnatal obesogenic environment (21, 22). In these studies, maternal obesity induced *via* a cafeteria-style HFD, lead to alterations in the central appetite circuitry and thus promotion of early onset obesity. Neonatal overnutrition by reducing litter size compounded the effects of maternal obesity *via* alterations in peripheral lipid and glucose metabolism that exacerbated the metabolic dysregulation observed in offspring (21). It has also been shown that maternal obesity interacts with a postweaning HFD to further amplify the adult programmed phenotype characterized by hyperphagia, adiposity, and metabolic dysregulation and was linked to increased hypothalamic NPY signaling and leptin resistance (23).

Of note, hypothalamic reductions in NPY expression and increased POMC expression can be induced by both maternal and neonatal overnutrition (21) with the greatest changes seen in those offspring of obese mothers who are then exposed to neonatal overnutrition *via* litter size reduction. Moreover, reduced hypothalamic NPY and AgRP expression shown in pups raised in small litters has been proposed to be an adaptation which overcomes the effects of neonatal overnutrition in early life (23).

A maternal HFD can stimulate proliferation of neuroepithelial and neuronal precursor cells in the hypothalamus during the embryonic period (24). This subsequently leads to a stimulation in the differentiation and proliferation of neurons and subsequent migration toward areas of the hypothalamus where consequently there is a domination of newly formed neurones that express orexigenic peptides (24). This increased neurogenesis, concomitant with hyperlipidemia, may therefore result in the longer term physiological and behavioral changes observed in offspring following weaning, including hyperphagia, preference for dietary fat, and increased body weights.

In addition to compromising the BBB (5), maternal obesity during pregnancy in the mouse can stimulate proliferation and thereby numbers of astrocytes in the fetal and early neonatal hypothalamus (particularly the ARH), which appears to be mediated, during fetal life, by interleukin (IL)-6 (25). Maternal obesity in the mouse can also lead to persistent synaptic impairments in offspring during development of the neuronal circuitry and may arise due to increased oxidative stress during the lactation period (26, 27). Leptin receptors are also expressed in mouse hypothalamic astrocytes with conditional deletion leading to changes in glial cell morphology and synaptic inputs onto those neurons involved in appetite regulation (28). Maternal obesity alters energy sensors (including mTOR and pAMPK) and epigenetic responses which modulate gene expression and neuronal differentiation and therefore can contribute to the obesity and related metabolic disorders, including hyperphagia, observed in offspring (29). Hyperleptinemia in the preweaning period arising from maternal obesity can induce lasting changes in the central melanocortin system (30) and melanocortin-4 receptor in the PVH appears to be a mechanism underpinning the programming of hypertension in later life (30).

There is a large variation in the composition of the experimental diets used across the range of rodent models and this has been of some debate over the robustness of the approaches used (e.g., the single fat source diets versus the “cafeteria” or “junk food” style diets) (31). In the rat, a maternal "junk food" diet can alter food intake preferences in offspring concomitant with programmed alterations in the mesolimbic (reward) pathways (32). Alterations in the fat:carbohydrate balance also suggest that even minor changes in macronutrient balance may alter phenotype outcome. As an example, when a high carbohydrate/HFD is utilized, insulin hypersecretion may induce compensatory mechanisms that may not manifest when HF/low-carbohydrate diets are used (33).

Evidence from existing models suggest that a maternal obesogenic environment facilitates central orexigenic pathways to induce obesity in offspring, while neonatal overnutrition *via* a reduction in litter size appears to be more closely associated with anorexigenic adaptations, possibly as a measure to counteract overnutrition (23). Further, there are compounding detrimental effects arising due to interactions between maternal obesity, neonatal overnutrition and postweaning HFD intake that amplify the metabolic disorders observed in offspring. Of note, some of the differences that have been observed across maternal obesity studies in which relate to key hypothalamic markers may relate to fed—fasting conditions at the time of analysis (i.e., differential responses to fed/fasted states) and differences in the dietary paradigm used, i.e., cafeteria versus single-source fat HFDs and length of dietary exposure(s). In the fed state, a reduction in NPY and increases POMC mRNA are observed in offspring from obese mothers (34). Under fasting conditions, the downregulation in NPY expression can be normalized to that of the controls in the offspring of obese mothers. Therefore, in a fasted state, NPY was not shown to be different in HFD-fed animals (34). As regards dietary composition, no direct comparisons have been made in maternal obesity models but it has been shown in non-pregnancy models that different obesogenic diets can elicit differential effects on ARC leptin receptor, NPY, and AgRP expression (35).

### Sheep

It is well established that the leptin peak present in the rodent during the early neonatal period parallels the normal development of central appetite regulatory regions, and alterations in the profile of the so-called “leptin surge” predisposes to obesity and metabolic dysfunction in offspring in later life. However, unlike humans and larger animal models including the sheep, rodents are born at an immature (altricial) stage of development so work undertaken in newborn lambs allows determination of the potential relevance to human development (36). Adult-like localization of leptin receptor (OB-Rb) gene expression and primary appetite regulatory neuropeptides in the hypothalamus are established prior to birth in the sheep. Thus, central appetite regulatory neural networks that have the potential to respond to changes in maternal nutrient supply are already present in the sheep fetus, which may impact on regulation of energy homeostasis both before and after birth (37). Long et al. have shown that maternal obesity can lead to a diminished neonatal lamb plasma leptin peak concomitant with increased plasma cortisol levels. It has also been shown that a maternal obesogenic environment is linked to increased fetal adiposity and altered expression of key enzymes involved in mediating fatty acid biosynthesis in adipose tissue (38). Similar results have been reported by Nicholas et al. whereby oocyte/embryo exposure to maternal obesity had lasting consequences for lipid metabolism in offspring (39). Recent work has also reported that maternal obesity leads to hyperphagia resulting in adiposity associated with adipocyte hypertrophy and increased fatty acid synthesis in adult male offspring of overnourished mothers although no potential central regulatory mechanisms were investigated (40). In the sheep, inducing maternal hyperglycemia or overfeeding leads to hyperinsulinemia, increase adiposity and increases in fetal POMC gene expression. These effects appear to be specific to the POMC system with AgRP, NPY, and cocaine- and amphetamine-regulated transcript peptide in the ARH unaffected by either exposure although the longer term effects on offspring phenotype are not yet known (41–43).

### Pig

Although a number of models of altered maternal nutrition have been established in the pig, there are little data on neuroendocrine readouts in the setting of maternal obesity. It has been shown that relative maternal undernutrition leads to piglets with an abnormal hypothalamic distribution of leptin receptors that is linked to altered food-intake behavior (44). This phenotype can be partially rescued *via* neonatal leptin administration. Maternal intake of a Western-style diet during pregnancy/lactation led to changes in microbiota profile, blood lipids, cognitive responses, and hippocampal neurogenesis in swine offspring (45). Recent work by Sanguinetti et al. has also shown that maternal HF feeding results in marked alterations in brain glucose metabolism in offspring thus predisposing to metabolic-neurodegenerative diseases (46–48).

### Rabbit

In the rabbit, as reported for other model species, offspring from mothers consuming a HFD are characterized by an adverse cardiovascular profile in later life which appears to manifest as a consequence of an altered hypothalamic sensitivity to leptin and ghrelin (47, 48). Offspring of HFD-fed rabbits displayed resistance to the anorexic effects of central leptin administration, less neuronal activation in the ARH, and PVN in HFD offspring compared with rabbits fed a maternal control diet. A HFD in the rabbit has also been shown to alter leptin and the melanocortin signaling pathway in the ventromedial hypothalamus (VMH) of offspring (49). This model was characterized by obesity-related hypertension which could be normalized following leptin receptor antagonist injections to the VMH.

### Non-Human Primate

Less work on the neuroendocrine programming of energy balance has been undertaken in the NHP. Maternal consumption of a HFD leads to increased anxiety-like behavior and perturbations in the serotonergic system in NHP offspring (50). Independent of maternal obesity, intake of a HFD during pregnancy alone can result in a widespread activation of proinflammatory cytokines (including IL-1β and IL-1 type 1 receptor) that may lead to changes in the melanocortin system. Abnormalities in POMC expression in the fetus, if persisting postnatally, can impact upon a number of peripheral systems, including body weight regulation, cardiovascular function, and altered stress responsiveness (51). Of note, dietary recuperation of obese NHP mothers to a standard healthy gestational diet, resulted in normalization of fetal melanocortin levels. Rivera et al. have also recently shown that both maternal HFD consumption and maternal obesity in the NHP during early life results in offspring that had a higher risk of becoming obese as a consequence of increased intake of palatable energy-dense food, a behavior that was associated with a reduction in central dopamine signaling (52).

## Gut–Brain Axis

Appetite can be controlled by food intake-regulatory peptides that are secreted from the gastrointestinal tract including ghrelin, glucagon-like peptide 1, peptide YY, cholecystokinin, and the recently discovered nesfatin-1 *via* the gut–brain axis (53). As key hypothalamic centers regulate appetite and body weight in response to hormonal and other central stimuli, dysregulation of gut–brain communication may underlie programmed metabolic disorders, including obesity.

The “hunger hormone” ghrelin is an appetite-regulating factor, primarily produced in the stomach, and is involved in regulation of energy homeostasis *via* actions in the ARH including effects on NPY and AgRP neurons. The hypothalamic ghrelin-sensitive circuits are dynamically regulated by central insulin and leptin concentrations (54). Ghrelin has been shown to mediate neural fiber growth in the ARH during the neonatal period and, in the neonatal mouse, alterations in ghrelin action during this critical developmental period may lead to increased adiposity in adulthood (55). Ghrelin antagonism results in enhanced ARH neural projections and lasting metabolic effects that include impaired leptin sensitivity and an increase in body weight, central adiposity, and blood glucose concentrations. In non-pregnant rats, work by Schele et al. has shown that central administration of ghrelin can influence food choice and, in accordance with its role as a gut–brain hunger hormone, appears able to alter food choice in an acute manner, with notable effects on promotion of a more "healthy" chow intake. The ventral tegmental area was proposed as a neurobiological substrate that likely accounted for these observed effects (56). Further work by Briggs et al. has shown that diet-induced obesity leads to ghrelin resistance as a consequence of reduced NPY/AgRP responsiveness to circulating ghrelin. This leads to suppression of the neuroendocrine ghrelin axis in order to limit further food intake (57). Altered ghrelin in the setting of maternal obesity has yet to be well defined although in the setting of neonatal overnutrition (*via* litter size reduction), pups display an impaired central response to peripheral ghrelin (58). Normalization of early hypoghrelinemia was relatively ineffective at correcting metabolic outcomes, suggesting ghrelin resistance in offspring raised in small litters.

Although the role of ghrelin has been well described broadly as a regulator of hypothalamic feeding pathways, little has been done as regards the potential differential effects of the two circulating forms of ghrelin; acylated versus des-acylated. Most research to date has focused on acyl ghrelin and the potential role of neonatal des-acyl ghrelin in developmental programming is not well defined. Recent work by Sominsky et al. has reported that early life overnutrition acutely affects ghrelin regulation in the short term, leading to a reduction in circulating des-acyl ghrelin and increased ARH expression of the growth hormone secretagogue receptor (59). These changes were paralleled by increases in neuronal activation in response to exogenous acyl, but not des-acyl, ghrelin in the ARH and PVN. Importantly, however, the observed effects on the ghrelin system arising from neonatal overnutrition did not persist into adulthood in the male offspring studied. Of note, these effects may be sexually dimorphic in nature as the same group has also recently reported that neonatal overnutrition leads to a persistent disruption of pituitary ghrelin signaling in females, potentially *via* exacerbating central stress responsiveness in these animals (60).

## Epigenetics and the Reward Pathways

Obesity and related metabolic disorders reflect a complex interplay of multiple factors, with interactions across numerous different genetic and environmental factors with modern obesogenic environments exacerbating the genetic risk for obesity. Fixed genomic variation appears to explain only a small proportion of obesity risk (61). As such, epigenetic modifications [including altered DNA methylation, changes in histone modifications and microRNAs (miRNAs)] offer alternative mechanisms by which alterations in the nutritional environment in early life may exert lasting phenotypic consequences in offspring.

Although a number of studies have investigated changes in epigenetic marks in the setting of maternal undernutrition, particularly around methylation of the leptin and POMC promoter, less has been described for maternal obesogenic models. Moreover, most studies to date have examined effects in peripheral tissues with a relative paucity of data in these models as relates to changes in neuroendocrine pathways. As an example, overnutrition during lactation can result in epigenetic modifications in key genes that are known to be involved in the insulin signaling pathway in skeletal muscle that can manifest as impaired insulin sensitivity in later life (62). Neuroendocrine alterations have also been reported in this model as pertains to insulin and leptin sensitivity with changes in hypothalamic insulin receptor and POMC gene methylation status (63, 64) and thus offspring phenotype is likely a consequence of epigenetic changes across processes that represent a complex and highly integrated network across several tissue systems. Hypermethylation of the POMC promoter persists in offspring of HFD dams, even when offspring are recuperated onto a standard chow diet postweaning. This places them at an increased risk for hyperphagia when challenged with a HF diet (65). These data also showed POMC promoter demethylation in mothers fed a HFD during pregnancy and lactation concomitant with a heightened decrease in body weight during lactation. A maternal HFD leads to hepatic cell cycle inhibition *via* alterations in cyclin-dependent kinase inhibitor P21 (Cdkn1a) activity and associated changes in gene expression and DNA methylation in neonatal rat offspring (66). However, cell cycle changes as relates to central processes were not investigated but are also likely perturbed given the potential role for P21 as a modulator of neurosecretory activity of hypothalamic neurons (67) and changes in neuronal secretion of P21 following alterations in food availability (68) and inflammation (69).

Work in NHPs has shown that, in addition to alterations in DNA methylation profiles, an energy-dense maternal diet can alter chromatin structure in the fetus *via* covalent modifications of histones (70). Further, a maternal HFD alters neonatal hepatic metabolism, albeit in a sex-specific manner, and in association with histone modifications, may contribute to the known sexual dimorphic responsiveness as regards changes in oxidative balance (71). Fetal sirtuin 1 (SIRT1) histone and protein deacetylase activity in NHPs can be modulated by a maternal HFD and suggests a role for SIRT1 in the mediation of the fetal epigenome/metabolome (72, 73). However, most studies have focused on hepatic readouts and whether similar histone changes occur in the neuroendocrine compartment has not been investigated but, as shown by Desai et al., are also likely to be perturbed with persistent changes in histone deacetylases observed in the ARC of offspring of obese mothers (29). miRNAs can also lead to histone modifications and changes in DNA methylation of promoters, which in turn affects the expression of target genes (74). In a sheep model of maternal obesity, miRNA expression in fetal muscle is altered (downregulation of miRNA let-7g expression) and therefore may represent a possible mechanism underlying the enhanced adipogenesis observed during development of fetal muscle (75). Whether there is a neuroendocrine contribution to this phenotype was not evaluated; given that the Let-7 family is highly expressed in the hypothalamus and proposed to be positively correlated with energy balance, further work is warranted as the central function of the Let-7 axis remains unclear (76).

In addition to homeostatic systems responsive to nutrient sensing and regulation of energy balance, maternal obesity can also perturb the hedonic system which includes stimuli related to reward and cognitive factors (4). In the context of early life developmental programming, exposure to HF/high-sugar diets has been shown to result in altered development of central reward systems, leading to increases in fat intake and altered responsiveness of the hedonic reward system to over consumption of energy-dense food in offspring in later life (32). As such, a maternal HFD can lead to altered gene expression and methylation of opioid and dopamine-related genes and thus may provide a further potential mechanism for programming of appetite control in offspring and a preference toward fatty/energy dense food intakes (70, 77). Obesity in mice at the time of conception itself has also been shown to program the opioid system in the offspring brain (77). Importantly for translative purposes, the reward pathways develops *in utero* in rodents with functional innervation in place by birth (78); investigation of the reward system may be therefore more translatable from rodent to the human than for some other processes (6).

Rats fed a “cafeteria-style” diet for even short periods of time display an impairment in sensory-specific satiety and these deficits persist even following dietary recuperation and withdrawal of the cafeteria-style diet (79). Further, feeding a cafeteria-style diet results in alterations in neuroadaptive responses that underlie reward systems with unrestricted intake of a highly palatable diet leading to an increase in the “reinforcing value of food” and weakened inhibitory control (79, 80). In addition, whereas ghrelin originally emerged as a gut-derived hormone involved in hunger and meal initiation and energy balance *via* effects on the hypothalamus, it also appears to have a role in reward-driven behaviors (81). This occurs *via* stimulation of the cholinergic–dopaminergic reward pathway *via* inputs to the ventral tegmental area and in the mesolimbic pathway, a circuit that is known to communicate the hedonic and reinforcing facets of natural rewards such as high fat/high sugar food. However, changes in these reward systems and the potential for ghrelin-mediated alterations in reward behavior have yet to be defined in the context of early life programming. Given that exposure to obesogenic diets during early life can impact upon the neurocircuitry that is involved in behavioral motivation (79), studies in the setting of maternal obesity and offspring behavior are clearly warranted.

## Interventions

A range of animal models have allowed for investigation of a number of experimental intervention paradigms (including pharmacologic and nutritional) aimed at reversing or ameliorating the effects of early life programming. A number of animal studies have shown that some adverse metabolic outcomes, manifest due to adverse developmental programming, can be rescued by targeted interventions during early life (82). It is important, however, to recognize potential sex-specific responsiveness to interventions and there is not a “one size fits all.” This was highlighted in the leptin intervention studies whereby sexually dimorphic responses to neonatal leptin treatment were reported (83, 84) and, importantly, early leptin treatment to male offspring of control pregnancies has the potential to induce an adverse metabolic phenotype in adulthood including hyperinsulinemia and increased adiposity (84). Further work also showed that the effects of neonatal leptin treatment on gene expression and DNA methylation in the livers of adult offspring appeared to be directionally dependent on maternal nutritional status although neuroendocrine changes were not examined (85).

### Leptin

Leptin has received significant attention as key mediator of developmental programming with alterations in the leptin in early life linked to an increased risk for obesity and metabolic dysregulation in later life (86). Leptin, under normal physiologic conditions, plays a critical role as a regulator of energy balance and acts centrally to control appetite and promote energy expenditure. Peripherally, it acts to reduce ectopic fat deposition, protect against lipotoxicity and to maintain glucose homeostasis.

Manipulations of leptin levels in early life in a range of model species utilizing leptin and/or leptin antagonists have highlighted a key role for leptin in the reprogramming of metabolic outcomes in offspring (86–89). During the “plastic” phase of early development (typically in rodents the first two weeks following birth), a number of studies have now clearly shown that maintenance of a critical leptin level is essential for the normal development of tissues and signaling pathways involved in metabolic control (86). As such, periods of relative hyper- or hypoleptinemia during this early developmental window may induce some of the metabolic adaptations which arise as a consequence of aberrant developmental programming. The mechanisms by which leptin influences the early developmental processes is still being defined and requires an understanding of the processes around the timing and amplitude of the early leptin surge, sex-specific effects, optimizing responsiveness to exogenous leptin treatment (i.e., critical windows), and a more in-depth understanding of leptins role in the development of the neurocircuitry involved in appetite control and energy homeostasis.

Most work to date using leptin as a programming “reversal agent” has been modeled in the rodent and there remains a paucity of evidence to support a primary role for leptin in the normal development and maturation of ARH wiring in higher model species (90). As an example, leptin levels are virtually undetectable in the NHP until the middle of the final trimester and parallel the late maturation of adipose tissue; this is evident even in fetal offspring from pregnancies characterized by obesity and hyperleptinemia (91). There may be a role for leptin in the refinement and branching networks of the ARH-NPY/AgRP projections closer to the time of parturition (92) but this has not been well characterized.

It must be noted however that, although leptin has received considerable attention as a key programming factor, work by Cottrell et al. has also provided evidence that programming of metabolic outcomes due to rapid catch-up growth *per se* does not require leptin signals (93). Using the *ob/ob* leptin deficient mouse model, low birth weight followed by rapid catch-up growth in the preweaning period resulted in an increase in body weight in adulthood characterized by hyperphagia and increased adiposity. These results indicated that factors independent of leptin were involved in the programming of energy homeostasis in this model. Further, recent work by Sominsky et al. showed that changes in leptin availability during early life influences development of hypothalamic networks in the short term but is partly resolved by adulthood (94). In this study, neonatal overfeeding led to an acute resistance of hypothalamic neurons to exogenous leptin and an early increase in AgRP/NPY fiber number which was resolved in adulthood. Neonatal leptin antagonism did not reverse the excess body weight or hyperleptinemia in the group overfed as neonates, thus suggesting factors other than leptin may contribute to the offspring phenotype (94).

#### Other Interventions

Work by Steculorum et al. in the mouse has shown that the uridine-diphosphate (UDP) receptor (P2Y6) signaling in AgRP neurons is involved in the initiation of obesity-associated hyperphagia and systemic insulin resistance (95). Central injection of UDP acutely promoted feeding in diet-induced obese mice and that pharmacological blockade of CNS P2Y6 receptors reduced food intake. These data suggest that P2Y6 may be a potential pharmacologic target to restrict both feeding and systemic insulin resistance in obesity and that P2Y6 receptor antagonism may have efficacy in reversing central programming effects in offspring induced as a consequence of maternal obesity.

It is now well-established that changes in the availability of dietary methyl donors can impact on the gene expression patterns by affecting DNA methylation at regulatory regions, a likely mediator for reprogramming effects developmental plasticity (96). In the non-pregnant state, methyl donor supplementation can reduce hepatic steatosis and modify the DNA methylation profile of fatty acid synthase (FAS) in rats fed an obesogenic diet (97). Maternal methyl supplements have recently been shown to exert lasting protective effects in offspring of HF-fed dams with changes in DNA methylation profiles of peroxisome proliferator activate receptor-γ, FAS, leptin, and adiponectin (98). Similarly, supplementation with methyl donors (including choline, betaine, folic acid, and vitamin B_12_) during the lactation phase can prevent the elevations in plasma homocysteine in offspring induced by a HF/high sugar intake in rat dams (99) and was linked to altered regulation of the methionine–homocysteine cycle. However, these studies have focused on peripheral tissues and yet to report on potential changes in the hypothalamic regions of interest. Work in the mouse has shown that maternal HFD consumption can lead to global and gene-specific decreases in DNA methylation in the brains of offspring; effects which are ameliorated with maternal methyl donor supplements although in a sex-specific manner (100).

## Discussion

The neural circuitry involved in the regulation of appetite and energy homeostasis has been extensively studied over the last two decades. However, although the anatomy and functional plasticity of the BBB has been well characterized, the molecular mechanisms regulating the interface and integration of peripheral metabolic signals to the ARH still remain poorly defined (101). A consensus has emerged that early life exposure to a maternal obesogenic environment increases the risk of developing obesity and metabolic disorders in adulthood. However, there is less agreement on the mechanisms through which such risk may be conferred (4). Collectively, data to date reveal that leptin, in particular, and ghrelin play key roles in facilitating the normal development of hypothalamic neural circuits and suggest that normal expression of these factors during the fetal/neonatal period is key for lifelong metabolic regulation (55, 86, 102). Although most work to date has been undertaken in the rodent where the early neonatal period is seen as the critical period for development of the neuroendocrine circuits, in many species the neuropeptide systems which control food intake develop and mature *in utero*, with large differences across the model species used (90).

Experimental animal data has provided some insights into mechanisms and critical windows; it also opens up more questions. As an example, how do alterations in maternal metabolic homeostasis alter the BBB interface in the ARH of offspring at birth? (101). Experimentally, further studies examining altered accessibility of circulating metabolic signals to ARH neurons at birth and/or the neonatal period during the period of neuronal plasticity to examine longer term impacts is also justified (101). Given that leptin, insulin, and ghrelin have all been shown to play a part in central reprogramming of neuronal wiring, does altering BBB access lead to development of central hormone resistance and disease in later life? Future work will also need to examine the transgenerational impacts of neuroendocrine programming following developmental exposures to a HFD through both maternal and paternal lineages (14). Although maternal HFD effects have been shown to extend through to the third generation (103), little is known re sex-specific programming effects and the central mechanisms involved. This raises a limitation of some experimental approaches whereby balanced experimental designs are not utilized thus precluding detailed examination of gene–environment interactions and further, sex-specific effects are often not examined. This is particularly true for intervention studies whereby it has evidenced shown that intervening in a replete environment may cause harm. An example of this is maternal taurine supplementation in a rat model of maternal obesity; supplementation to obese mothers decreased the high neonatal motility observed in this group but increased neonatal mortality in offspring of supplemented control dams, possibly due to the hypoglycemic effects of taurine (104). As detailed above, similar outcomes have also been reported for neonatal leptin treatment where offspring outcomes are directionally dependent on maternal nutritional background and offspring sex (83–85).

Circulating hormones integrate and impact upon multiple components of hypothalamic development and therefore play a key role in the coordination of neural circuit formation and normalization of neuroendocrine signaling pathways. It is likely therefore that deficiencies in any of these factors during sensitive periods of developmental plasticity can lead to lasting structural and functional consequences. As detailed above, in model species that give birth to mature young (e.g., NHP, sheep) maximal brain growth and a large proportion of neuroendocrine maturation takes occurs in fetal life (105, 106). Rodents are born immature and thus hypothalamic maturation is initiated prenatally but not finalized until the second week of postnatal life (107). Maternal lactational capacity therefore is a primary determinant of nutrient supply in the rodent rather than dependence upon the nutrient transfer capacity of the placenta. Alterations in the composition of milk during the suckling phase therefore have the potential to exert marked effects on hypothalamic maturation that can be mediated through changes in milk hormone concentrations. For example, rat neonates born to obese mothers exhibit a precocious surge in circulating insulin paralleled by abnormalities in the neonatal leptin surge (amplified and prolonged) with concomitant elevations in leptin mRNA expression in visceral adipose tissue (19). It was hypothesized that extended release of high leptin concentrations by neonatal rats born to obese mothers resulted in leptin resistance and permanent effects on hypothalamic function involving the ARH and PVN and therefore may underlie the later hyperphagia and obesity observed in these animals. These effects are likely mediated in part by alterations in milk composition with a maternal HFD in the rat shown to be associated with increased milk protein and lipid concentrations (108, 109). These changes in compositional profile result in neonatal overnutrition and can result in thyroid and adrenal dysfunction and increased adiposity in male offspring as early as weaning (110). However, despite the well-characterized developmental differences across the range of model species used in programming studies, both precocial and altricial species appear to exhibit several potential commonalities in programming mechanisms that result in the altered adult phenotype, particularly as regards alterations in hypothalamic wiring.

Although epigenetic mechanisms can mediate the effects of adverse maternal obesity during pregnancy/lactation and offspring developmental malprogramming, it has also been suggested that increases in accessibility of ARH neurons to peripheral metabolic signals could arise *via* processes similar to that seen with the adaptive responses to fasting (111). The BBB restricts delivery of circulating factors that transfer metabolic information to the neural networks that regulate energy homeostasis. During fasting, the decrease in blood glucose levels alters the structural organization of the blood–hypothalamus barrier and results in the improved access of metabolic substrates to the ARH (111). These data that glucose plays a role in the control of blood–hypothalamic exchange through a vascular endothelial growth factor (VEGF)-dependent mechanism and demonstrated a role for tanycytes and associated permeable microvessels in the adaptive metabolic response to fasting. This mechanism could also translate to strategies for intervention—treatment of offspring of obese mothers with VEGF receptor inhibitors could prevent the deleterious effects of maternal obesity by altering capillary fenestration of the median eminence branches extending to the ARH.

Wherever possible, animal models of maternal obesity should be designed based on the principals recently developed *via* the Animals in Research: Reporting *In Vivo* Experiments guidelines (112). This includes detailed reporting of the species/strain/sex of the animals used, balanced experimental design, detailed dietary composition details and implementation of processes around randomization and blinding to reduce experimental bias in animal selection. Such approaches will allow increased relevance of studies undertaken in the setting of maternal obesity and increase translative capacity to the human setting. As above, many studies to date have not examined the impact of sexual dimorphic responses to different programming stimuli and these differences need to be acknowledged as they inform on the mechanistic underpinnings of how divergent phenotypes emerge. Adoption of a life course approach to examine phenotype development allows early identification of related risk markers (113, 114), with the potential that nutritional, pharmacological and lifestyle (e.g., exercise) interventions may aid in the amelioration of non-communicable diseases, particularly in those developing societies that are in the process of nutritional transition. The different animal models utilized will have different utility as regards translation to the human condition. It is noteworthy however, that despite the differences in timing of neurodevelopmental processes across the models species used and the differences in dietary compositions and duration of exposures, the phenotypes reported often mirror those seen in the clinical setting of the metabolic syndrome. This speaks to the validity of the range of animal models currently used to investigate the neuroendocrine mechanisms underpinning developmental programming. As perinatal manipulations can permanently alter the systems involved in appetite control and energy homeostasis, there is an urgent need to identify the key factors responsible as a means of stemming the worldwide obesity epidemic *via* effective targeted intervention strategies during critical early periods of developmental plasticity.

## Author Contributions

MV wrote the first draft of the review and then was further revised and updated by SS and CR.

## Conflict of Interest Statement

The authors declare that the research was conducted in the absence of any commercial or financial relationships that could be construed as a potential conflict of interest. The reviewer LD and handling editor declared their shared affiliation.
